# Nuclear trafficking of secreted factors and cell-surface receptors: new pathways to regulate cell proliferation and differentiation, and involvement in cancers

**DOI:** 10.1186/1478-811X-4-7

**Published:** 2006-10-18

**Authors:** Nathalie Planque

**Affiliations:** 1 Laboratoire d'Oncologie Virale et Moléculaire, Université Paris7-Denis Diderot, UFR de Biochimie, 2 place Jussieu, 75005 Paris, France

## Abstract

Secreted factors and cell surface receptors can be internalized by endocytosis and translocated to the cytoplasm. Instead of being recycled or proteolysed, they sometimes translocate to the nucleus. Nuclear import generally involves a nuclear localization signal contained either in the secreted factor or its transmembrane receptor, that is recognized by the importins machinery. In the nucleus, these molecules regulate transcription of specific target genes by direct binding to transcription factors or general coregulators. In addition to the transcription regulation, nuclear secreted proteins and receptors seem to be involved in other important processes for cell life and cellular integrity such as DNA replication, DNA repair and RNA metabolism.

Nuclear secreted proteins and transmembrane receptors now appear to induce new signaling pathways to regulate cell proliferation and differentiation. Their nuclear localization is often transient, appearing only during certain phases of the cell cycle. Nuclear secreted and transmembrane molecules regulate the proliferation and differentiation of a large panel of cell types during embryogenesis and adulthood and are also potentially involved in wound healing. Secreted factors such as CCN proteins, EGF, FGFs and their receptors are often detected in the nucleus of cancer cells. Nuclear localization of these molecules has been correlated with tumor progression and poor prognosis for patient survival. Nuclear growth factors and receptors may be responsible for resistance to radiotherapy.

## Background

The classical view of the way secreted molecules such as growth factors and protein hormones operated was that they acted at the cell surface by binding membrane receptors and activating cascades of intracellular second messengers, leading to the regulation of expression of specific target genes. Their internalization in endosomal vesicles and degradation in lysosomal compartment was seen as a way to stop their activation (For further details, see review [1]). But some evidence showed that their modes of action seem to be more complex. An unexpected finding was that internalized Fibroblast Growth Factors (FGFs) can have a long life inside the cell (over 24 hours), and it has been shown that several FGFs such as FGF1, FGF2 and FGF3 can act both extracellularly and intracellularly.

Nuclear localization of FGFs and Epidermal Growth Factor (EGF) and of their cell surface receptors (FGFRs and EGFR respectively) has been well documented over the last 15 years in normal and physiopathological states. The deciphering of the underlying mechanisms of such apparently unexpected subcellular localization revealed that secreted factors and their receptors were internalized into the cytoplasm and routed to the nucleus, where they exert diverse functions such as regulation of gene transcription. By modulating the expression of genes involved in cell cycle progression, nuclear forms of growth factors (GFs) and of their surface tyrosine-kinase receptors (RTKs) often regulate cell proliferation. Other RTKs such as Vascular Endothelial Growth Factor (VEGF) and Nerve Growth Factor receptors have also been detected in the cell nucleus. Several lines of data also reported nuclear trafficking for a variety of other classes of secreted molecules such as interleukines and hormones. For instance, Interleukin-1 (IL-1), IL-5, Interferon-γ (IFNγ), Growth Hormone (GH), Prolactin, Lactoferrin, Insulin-Like Growth Factor Binding Proteins (IGFBPs) have been detected in the cell nucleus (reviewed in [2]).

The number of secreted proteins concerned by nuclear trafficking is growing. Emerging data on members of the CCN family enlarge the circle of secreted factors that are involved in this phenomenon [3-5]. The CCN proteins are secreted factors that act as key regulators in embryonic development, and are associated with severe pathologies including fibrotic diseases and cancers [6-12]. Acting on cell adhesion, migration, proliferation, differentiation and survival, they regulate fundamental cell processes of fetal and adult life, such as angiogenesis, skeletal development, wound repair and inflammation. The CCN family is composed of 6 members in human, which have been grouped on the basis of structural analogies. The CCN proteins are indeed composed of four structural modules that contain regions homologous to sequences found in IGFBPs, Thrombospondin and Von Willebrand Factor (Figure 3A). The carboxy-terminal domain (CT) contains cysteine residues that could form a cystine knot.

The mechanisms of transport from the extracellular compartment to the cell nucleus is well understood for FGFs, EGF and their RTKs. By contrast, transportation to the nucleus of the other secreted molecules is still largely unknown. Secreted and transmembrane proteins are generally internalized by endocytosis, delivered in the cytoplasm, and then transported to the nucleus by classical pathways involving specific proteins (importins) that recognize nuclear localization signals (NLS) in cargo proteins. This current review will not cover in detail the nucleocytoplasmic shuttling mechanisms since the recent literature presents detailed reviews about this aspect [13-15]. Instead, we will focus on nuclear functions of secreted proteins and cell surface receptors in normal and pathophysiological states.

## Nuclear shuttling

### Internalization from the plasma membrane

When reports started to appear on the nuclear localization of GFs and surface receptors, one of the first questions addressed was to determine if these molecules came from the cell surface after internalization or if they were alternative intracellular forms lacking the signal peptide of secretion. Intact, full-length molecules have been found in the nucleus. According to the current literature, it has become clear that nuclear cell-surface receptors and ligands often come from the cell surface. Macromolecules can be internalized by two major endocytic pathways, involving either caveolin or clathrin.

FGF growth factors not only activate transmembrane receptors but may also be co-internalized with their receptors in the cytoplasm and translocated to the nucleus. FGFs bind to 4 transmembrane receptors (FGFR1–4) expressed in a tissue-specific manner. Several approaches using elegant methods revealed recently that nuclear FGFRs and FGF1 and FGF2 are first internalized from the cell surface by endocytosis. These processes seem to be clathrin-independent, but may involve lipid/caveolin dependent mechanisms. Furthermore, delivery of exogenous FGFs from intracellular vesicles into cytoplasm requires establishment of an electrical potential across vesicular membrane, involving proton pump and Na^+^/K^+ ^– ATPase [16,17]. These processes have recently been reviewed [1,2,18]. PI3 kinase activity is required for translocation of FGF1 and FGF2 across the endosomal membrane [17], as well as Heat Shock Protein-90 (Hsp90) [19]. Interestingly, transport of FGF1 and FGF2 across endosomal membranes seems to be most efficient during the G1 phase of the cell cycle [17].

The use of labeled EGF showed that nuclear EGF was derived from extracellular added ligand. EGFR belongs to the ErbB family of transmembrane tyrosine-kinase receptors, which encompasses four structurally members (ErbB1–4) also known as HER. ErbB/HER receptors bind specific secreted molecules, with the exception of ErbB2 (a.k.a. Neu), that has no obvious ligand. EGFR and ErbB2/HER-2 are translocated from the cell surface to the nucleus through endocytosis [20,21]. Blocking EGFR endocytosis suppresses its nuclear import [22], and EGFR nuclear shuttling is dependent on EGF stimulation.

IGFBP2, IGFBP3 and IGFBP5 have been detected in the cell nucleus. Furthermore, nuclear import of endogenous IGFBP3 was shown to require IGFBP3 secretion and re-uptake. Endocytosis of extracellular IGFBP3 is mediated by caveolin-pathway and a clathrin-pathway specific to transferrin/transferrin receptor [23].

Externally added CCN2 recombinant protein has been found to enter the cell and localize in the perinuclear compartment [4]. CCN proteins physically interact with several classes of cell-surface proteins such as integrins, Heparan Sulfate Proteoglycans (HSPGs), Notch and connexins. Though a CCN2-binding protein has been identified in the plasma membrane of HCS-2/8 chondrocytes, definitive clues about the existence of transmembrane receptors specific to CCN proteins are still lacking to date. The mechanism of cell entry is completely unknown.

### Translocation to the nucleus

Nuclear import via classical NLS pathways seems to be a general feature for secreted factors and their receptors. Briefly, small molecules (size of less than 40 kDa) can diffuse freely through the nuclear pores, but nuclear import generally implicates basic amino-acids-rich NLS sequences, which are recognized by carrier proteins of the importin (IMP) family. The prototypic carrier consists of 2 importin sub-units: IMPα, that contacts the NLSs in the cargo proteins that have to be imported to the nucleus, and IMPβ1, that binds to hybrophobic repeat sequences in nucleoporins proteins (Nups), that constitute the nuclear pore complexes (NPCs). Some cargo proteins can be transported to the nucleus by IMPβ1 alone. For further details on nuclear shuttling, see reviews by [13-15].

The nuclear import of IGFBP3 and IGFBP5 is dependent on a NLS pathway mediated by IMP-β [24]. The Parathyroid Hormone-related Protein (PTHrP), a secreted protein that is related to the parathyroid hormone, a major regulator of calcium homeostasis, also contains a NLS. Several types of NLS can be found in FGFs molecules. FGF1 and FGF2 present different isoforms of low and high molecular weight (LMW and HMW respectively), resulting from alternative translation initiation. LMW FGFs are secreted and need to be re-internalized to be routed to the nucleus. In contrast to LMW isoforms, HMW forms (21–34 kDa) are not excreted from the cell and are routed to the nucleus directly from the cytoplasm. Both LMW and HMW FGFs contain one or several NLSs. For instance, a secreted form of FGF1, that does not contain a classical signal peptide of secretion, possesses two NLS: one located in its amino-terminal part and the other, a non-conventional bipartite NLS, in its carboxyterminal extremity [25]. Both are important for efficient transport of FGF1 to the nucleus. LMW FGF2 contains a signal peptide for secretion and also shows a non classical bipartite NLS in its C-terminal part [26,27]. Stimulation of FGFR1 by FGF1 and FGF2 results in the nuclear translocation of FGFR1 in an IMP-β dependent manner [2,28]. HMW FGFs show an Arginine-rich NLS-like in their aminoterminal extension. Along the same lines, a subfamily of FGF proteins, FGF11–14, named FHFs (for FGF Homology Factors) are devoid of signal peptide but contain a sequence rich in basic amino acids that resemble a NLS. They are not secreted. FGF11 was even shown to accumulate in the nucleus in NLS-dependent manner (reviewed in [1]).

FGF3 constitutes an interesting case. A duality between secretion and nuclear import has indeed been reported for this growth factor. FGF3 contains three polycationic sequences that are important for nuclear localization. The first one is a bipartite NLS located in the amino-terminal part of FGF3, and the other two were found in the carboxy-part of the molecule. These NLS exert additive effects in counteracting the signal peptide [29,30]. Secreted and nuclear forms of FGF3 exhibit opposite effects on cell proliferation.

**Figure 1:**
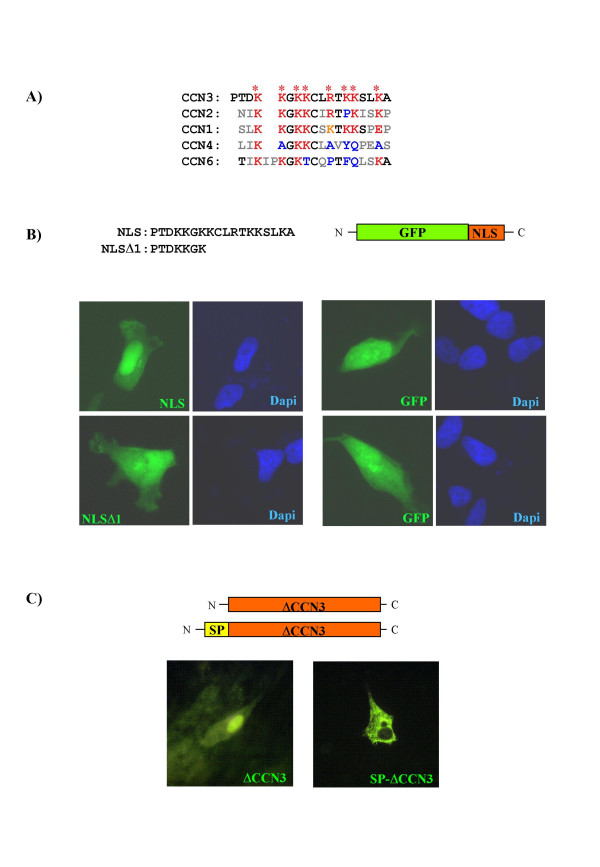
**A NLS is located in the CT domain of the CCN3 protein**. **A) **Conservation of the primary structure of this Arg/Lys-rich NLS in the human CCN proteins at the beginning of CT module. Basic amino acids are colored in red and marked with an asterisk in the human CCN3 sequence. These Arginine and Lysine residues are colored in blue when they are not conserved in the other CCN proteins. Replacement of a basic residue by another is represented in orange. Other non-conserved amino acids are colored in grey. **B) **Human G59 glioblastoma cells transiently transfected with GFP-NLS constructs. **C) **Baby Hamster Kidney 21 (BHK21) cells transiently transfected with CCN3 constructs (similar strategy of construction as the one described in [5]. GFP and DAPI autofluorescence were detected with epifluorescence under appropriate filters. CCN3 was detected by immunostaining with K19M antibodies [122].

An amino-truncated form of CCN3 devoid of signal peptide has been detected in the nucleus of cancer cells ([3,31,32] and unpublished data). The CCN3 protein was recently shown in our laboratory to contain a NLS that targets intracellular forms devoid of signal peptide to the nucleus [5]. CCN3 NLS is a polycationic sequence rich in lysine (PTDKKGKKCLRTKKSLKA) located at the beginning of the CT module. This NLS is sufficient to drive the Green Fluorescent Protein (GFP) to the nucleus ([5] and Figure 1B). These observations suggest that CCN3 forms are transported to the nucleus via importins pathway. It is worth noting that the PTDKKGK sequence does not seem to be able to drive GFP to the nucleus (Figure 1B), though it has been identified as a NLS, using a bioinformatic approach (PSORT II server: [33]). The CCN3 signal peptide seems to be dominant on the NLS. Forms that contain both the signal peptide and the NLS are localized at the cell surface ([5] and Figure 1C). An amino-truncated CCN3 protein has been found in the supernatant of cell producing the full length secreted form of CCN3 [34]. The most attractive hypothesis about the generation of nuclear truncated forms of CCN3 is that, after secretion of the full-length protein and cleavage in the extracellular compartment, the carboxy-terminal part of the protein could re-enter the cell and be routed to the nucleus via an NLS-dependent pathway [5,7]. The basic amino-acids of the CCN3 NLS are highly conserved in CCN2 and CCN1 (Figure 1A). This NLS sequence may therefore be responsible for nuclear transport of exogenous CCN2 after internalization from plasma membrane. Along the same line, CCN1 forms may also be routed to the nucleus, though no evidence is documented in the current literature. By contrast, the poor conservation of this Arg/Lys-rich sequence in CCN4 and CCN6 suggest that these two proteins would not be routed to the nucleus, or at least not by this mechanism.

IFNγ contains a prototypic NLS. Mutations in the IFNγ NLS abolish its biological activities. Endocytosed IFNγ binds to IMPα5, suggesting a nuclear import via the IMPα/β1 dependent pathway. The α-subunit of the heterodimeric IFNγ receptor (IFNGR-1) also translocates to the nucleus in IFNγ-treated cells. After internalization by endocytosis, IFNγ/IFNGR-1 complexes are translocated to the nucleus in association with Signal Transducer and Activator of Transcription-1a (STAT1a). STATs are transcription factors activated by a large panel of secreted proteins (cytokines, hormones, etc). Inactive STATs are localized in the cytoplasm and translocate to the nucleus after phosphorylation by a RTK. STAT1a is able to directly contact IMPα5 via an unconventional NLS that exhibits a much lower affinity for importins than conventional NLSs. These observations lead Johnson and his colleagues, in a recent review [13], to propose an attractive hypothesis whereby the nuclear transport of the IFNγ/IFNGR-1 complex brings STAT1a into the nucleus and allows IFNγ-specific responses by a downstream effector, that is besides being activated by plethora of secreted factors.

In contrast to the above quoted examples, PRL lacks a NLS sequence, and its nuclear translocation requires the presence of its receptor at the plasma membrane [35,36]. The Cyclophilin B protein (CypB), that contains a putative NLS, has been proposed to transport PRL to the nucleus [37]. Related to PRL, GH exhibits exactly an inverse situation. Indeed, GH, its cell surface receptor (GHR) and a related protein (GHBP) that binds GH have been found in the nucleus but this time, nuclear translocation of GHR and GHBP requires GH stimulation.

Similar to PRL, EGF does not contain an intrinsic NLS and its nuclear localization is dependent on expression of surface EGFR, that has a polycationic NLS. EGFR interacts with IMPα1/IMPβ1 [22]. Binding of EGF to nuclear EGFR results in autophosphorylation of EGFR and phosphorylation of other nuclear proteins (reviewed in [13]). Intriguingly, the Schwannoma-derived Growth Factor (SwGF), another ligand for EGFR, does contain a NLS. Other RTKs of the EGFR/ErbB family are also found in the nucleus. For instance, ErbB2 is driven to the nucleus in an IMPβ-dependent pathway [21]. ErbB4/HER4 contains three potential polycationic NLS in its carboxy-terminal part, but only one of them was shown to drive the GFP protein to the nucleus ([38]. Of note, nuclear forms of Erb4/HER4 do not come from internalization of the transmembrane form by endocytosis. Instead, transmembrane Erb4/HER4 is cleaved by the metalloproteinase γ-secretase at the plasma membrane, releasing its cytoplasmic tail which is translocated to the nucleus [39,40].

**Figure 2:**
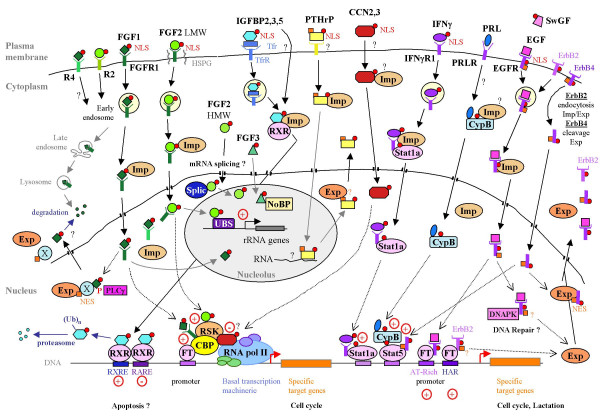
**Summary of the diverse pathways of nuclear shuttling and nuclear functions of secreted proteins and cell surface receptors**. Trf: transferrin; Imp: importin; Exp: exportin; RNA PolII: RNA polymeraseII; (Ub)_n_: polyubiquitination; Splic: splicing machinery; UBS: upstream factor binding site; FT: transcription factor (For nuclear EGFR, the E2F1 and STAT3 transcription factors were identified as binding partners).

All the above examples depict the diversity of situations in which secreted proteins and their cell surface receptors are internalized from the plasma membrane or directly routed to the nucleus from the cytoplasm. A summary of the various cases is presented in Figure 2. Once they have acted in the nucleus, secreted and cell-surface molecules have to be inactivated.

**Figure 3:**
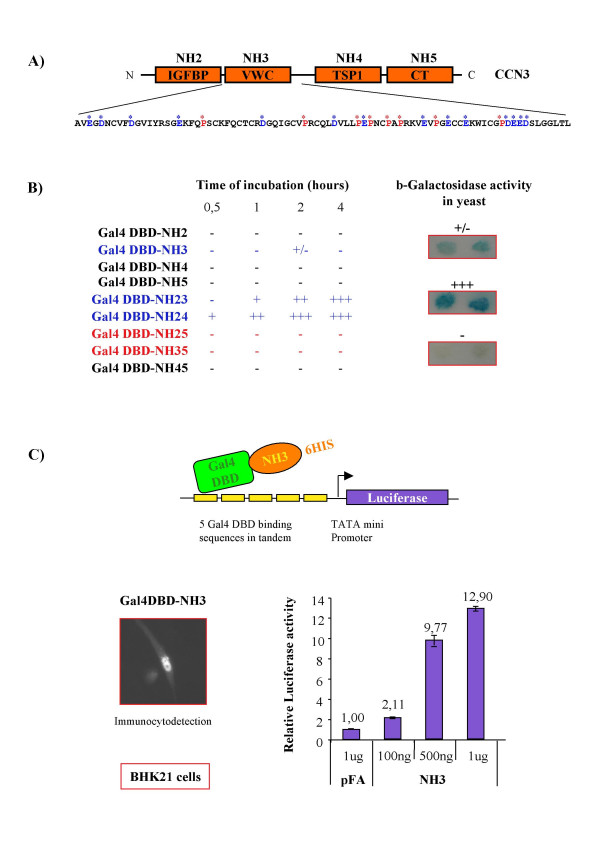
**A transactivation domain is located in the VWC module of the CCN3 protein**. **A) **Nomenclature and schematic depicting of the modular structure of the CCN3 protein. IGFBP: Insulin Like Growth Factor Binding Protein-like module; VWC: Von Willebrand factor-like module; TSP1: Thrombospondin-like module; CT: C-terminal module. Acidic residues are colored in blue, proline in red, and are marked by an asterisk in the human CCN3 VWC module. **B) **The pGBT9 transfectants (Gal4DBD fusion proteins) were selected and grown in minimal medium deprived of tryptophane. The Y190 recipient yeast used in this experiment contains a recombinant lacZ reporter gene cloned downstream a promoter containing upstream Gal4 DBD binding sites (activator binding sites). Qualitative assays were performed on Whatman filter paper. The β-galactosidase activity was monitored every half hour for a total period of 8 hours. **C) **Upper panel: diagram depicting the mammalian reporter system used in this study (pFA/pFR-Luc, Stratagen). Lower panel left: Immunocytofluorescent detection of Gal4DBD-NH3 protein in transiently transfected BHK21 cells, showing nuclear localization of the fusion protein using anti-His antibody (protocol for immunodetection described in [5]). Lower panel right: BHK21 cells were co-transfected with 1 μg of pFR-Luc and increasing amounts of NH3. The total amount of DNA used for each transfection was kept constant by adjustment with pFA DNA. BHK21 (baby hamster kidney 21) cells were grown and transfected as decribed in [5]. Posttranfection (48 h), luciferase and β-galactosidase activity from cell lysates was measured as described in [5].

### Export from the nucleus and inactivation in the nucleus

The regulation of the nuclear functions of these proteins begins to be deciphered. Nuclear activities of GFs can be regulated by export from the nucleus to the cytoplasm. Recent reports indeed showed that the nucleocytoplasmic shuttling of these proteins involves classical nuclear export pathways (review on nuclear export mechanisms in [15]). Active nuclear export generally implicates a leucine-rich nuclear export signal (NES) in the cargo proteins. This NES is recognized by exportin (EXP) proteins, which allow nuclear pore crossing.

ErbB4/HER4 contains such an NES. Nuclear export of EGFR, ErbB2/HER2 and PTHrP is blocked by leptomycin B, an inhibitor of CRM1/EXP-1 protein [21,22,41], suggesting that they are also exported by NES pathways. A phosphorylation process regulates nucleocytoplasmic shuttling of FGF1. Externally added FGF1 is phosphorylated in the nucleus by PKCδ and subsequently rapidly exported to the cytoplasm, in a manner that is inhibited by leptomycin B, then dephosphorylated and probably degraded in the cytoplasm [42]. Non phosphorylated FGF1 remains in the nucleus. FGF1 does not contain an obvious NES. It is therefore possible that it is exported from the nucleus by binding to a partner that possesses a NES (Summary in Figure 2).

Alternatively, some nuclear secreted proteins and transmembrane RTKs may be inactivated inside the nucleus by proteolysis, as suggested by the recent discovery of ubiquitin/proteasome-mediated degradation of nuclear IGFBP3 [43] (Figure 2).

## Regulation of gene transcription and other nuclear functions

According to the current literature, it appears that nuclear secreted factors and RTKs generally regulate gene transcription. Though initial studies depicted binding of nuclear RTKs and GFs to DNA, no structural DBD has ever been characterized in these molecules. Therefore, it is likely that they do not directly bind DNA, but rather interact with DNA binding partners, and that their transcriptional activity depends on interactions with other transcription factors such as specific transcription factors and general co-regulators. Recent studies also revealed potential roles in other nuclear processes such as DNA repair and RNA processing.

### Interaction with transcriptional regulators

EGFR and EGF were initially shown to bind chromatin, and SwGF, to AT-rich DNA sequences [44,45]. We now know that some nuclear RTKs of the EGFR/ErbB family specifically bind target promoters. For instance, nuclear EGFR binds to AT-rich sequences in the promoter of the *Cyclin D1 *gene and activates its transcription [46]. Likewise, ErbB2/HER2 binds to a specific sequence called HAS (HER2 associated sequence). The promoters of the *Cyclooxygenase2 *(*COX2*), *PRPK*, *MMP16 *and *DDX10 *genes were identified as direct target promoters of nuclear ErbB2 [47]. It is worth noting that positive correlations have been established between nuclear localization of EGFR/ErbB receptors and expression levels of target genes in tumors (see below), underlining the pathophysiological relevance of these results. Nuclear EGFR complexes with STAT3 and co-regulates transcription of the *iNOS *gene [48]. In a similar manner, its interaction with E2F1 activates the expression of the *b-Myb *gene, which encodes an essential transcription factor for cell cycle progression [49]. EGFR-E2F1 association with the *b-Myb *promoter is only detected during the G1/S phase transition [49]. Interestingly, inhibition of major EGFR downstream pathways such as PI-3K and ERK does not significantly suppress the EGFR-induced *b-Myb *expression [49]. Similar to nuclear EGFR, ErbB4/HER4 co-activates the expression of the *β-casein *gene by binding STAT5a [38]. ErbB4 may therefore potentiate the expression of milk genes by STAT5a during pregnancy and lactation. Similarly, nuclear PRL-CypB and GHBP potentiate STAT5 transcriptional activities [37,50].

As for the EGF/ErbBs family, members of the FGFs/FGFRs family regulate the transcription of specific target gene by interacting with various nuclear partners. FGFR1 transcriptional activities were suggested when immunoelectron microscopic, and confocal analysis revealed that FGFR1 co-localized with transcriptionally active chromatin [51]. FGFR1 binds to the general co-activator CREB-binding protein (CBP) and up-regulates the expression of specific target genes such as *FGF2 *and *Tyrosine Hydroxylase *by increasing the recruitment of RNA polymerase II and histone acetylation at active promoters [51,52]. Nuclear FGFR1 also physically interacts with Ribosomal S6 Kinase isoform 1 (RSK1), a regulator of CBP and histone phosphorylation, and regulates its transcription activities toward transcriptional regulators complexes [53]. The *Neurofilament-L*, *neuron-specific enolase microtubule associated protein-2 *(*MAP2*) and *c-jun *genes are now identified as target genes of nuclear FGFR1 [28,51]). Nuclear FGFR1 also potentiates *cyclin D1 *expression [28]. Nuclear FGFR1 thus regulates the expression of genes that are involved in cell growth and differentiation. Interestingly, as for EGFR, the transactivation of the target genes of nuclear FGFR1 is not induced by stimulation of cell-surface FGFR1, suggesting that the functions of a RTK at the plasma membrane may differ from the ones inside the nucleus.

Initially, externally added FGF2, which accumulated in the nucleus, was shown to correlate with stimulation of ribosomal gene transcription, and activate transcription in cell-free system [54-56]. More recently, in GST pull-down assays, nucleolin, histone H1, Upstream binding factor (UBF), an essential transcription factor for rRNA transcription, and ribosomal protein P0 were found as LMW FGF2 interacting partners [57]. Furthermore, LMW FGF2 bound to UBF associates with rRNA genes and regulates rRNA transcription both *in vitro *and *in vivo *[57]. Taken together, these results suggest a major role of nuclear LMW FGF2 in the regulation of rRNA genes. In addition, nuclear FGF2s regulate the transcription of genes transcribed in mRNAs. Indeed, the *phosphoglycerate kinase 1 *and *2 *genes are regulated by nuclear FGF2 in a promoter-specific manner [56]. Exogenously added LMW FGF2 enter the nucleus and directly interacts with RSK2 [58], another isoform of RSK, in a cell cycle-dependent manner. Nuclear LMW FGF2 may therefore potentiate the RSK2 activities during the cell-cycle progression (see below). Nuclear HMW FGF2s regulate the promoter activity of the *IL-6 *gene [59], and as FGFR1, they stimulate the transcription of the *Tyrosine Hydroxylase *gene via cAMP Response Element (CRE) sequences [51]. HMW nuclear forms of FGF2 were also shown to physically interact with the anti-apoptotic putative transcription factor FIF2 [60]. This interaction may be important for cell response to stress.

Preliminary data suggested that nuclear CCN proteins may regulate transcription. CCN2 activated transcription in a cell-free system [4], and the CCN3 CT module was found bound to a specific NFκB-like sequence in the promoter of the *Plasminogen Activator Inhibitor-2 *(*PAI-2*) gene [61]. Intriguingly, no functional activity for this binding has been identified to date. We were unable to detect any variation of the transcription level of the luciferase gene cloned downstream human *PAI-2 *promoter sequences when co-transfected with various nuclear forms of CCN3 (data not shown and [5]). However, we found that CCN3 was able to modulate transcription in other systems. We used a single hybrid system in yeast, in which plasmids expressing CCN3 recombinant proteins fused to the DBD of the yeast Gal4 transcription factor. The recipient yeast used for transient transfections contained the *lacZ *reporter gene cloned downstream a promoter containing Gal4-DBD binding sites. The results reported in Figure 3B (Li et al. unpublished) indicated that under conditions in which the full-length CCN3 protein showed no activity, a CCN3 recombinant protein containing only IGFBP, VWC, and TSP1 modules (NH24) (nomenclature depicted in Figure 3A), induced a strong transcription transactivation. Similar results were obtained with pACT2-derived constructs, which are known to express higher levels of recombinant protein than pGADGH. The use of plasmids expressing either individual domains or combinations of domains, allowed us to establish that the VWC module of CCN3 was responsible for the transactivation activity of the recombinant proteins. Indeed, clones containing the VWC module (NH3, NH23, and NH24) were positive in the β-galactosidase assay, whereas NH2, NH4, NH5 and NH45 were negative. The negative results observed with NH35 (containing the VWC, TSP1 and CT modules) and with the full-length CCN3 (NH25) raised the possibility that the presence of the CT module was interfering with the transactivating activity of the VWC module. These results are in agreement with the transinhibitory effect of the CT module that we have described in a recent report [5].

In parallel, quantitative assays were performed in mammalian cells that confirmed the previous results obtained in yeast. We used a pFA-CMV plasmid that expressed the VWC module of CCN3 fused in frame with the DBD of Gal4 (see [5] for the construction strategy). A six histidine tag (6HIS) was added at the C-terminal part of the fusion protein to allow immunological detection of the recombinant proteins (Figure 3C, lower panel left). For the transactivation assays, cells were co-transfected with the pFA-NH3 expression plasmid, the pFR-Luc reporter plasmid, in which the luciferase gene transcription was under the control of 5 Gal4 binding sites, and a lacZ vector for β-galactosidase normalization. The results established that the VWC module could transactivate transcription in mammalian cells in a dose-response manner (Figure 3C, lower panel right). TADs are generally rich either in basic amino acids, or in proline. CCN3 VWC module encompasses both several basic and proline residues (Figure 3A). The IGFBP and TSP1 modules exert no significant effect on transcription (not shown), whereas the CT module contains an inhibitory domain of transcription that is stronger than the transactivation domain located in the VWC module [5]. CCN3 nuclear forms may thus act as transcriptional repressors [5]. Therefore, using a Gal4 reporter system, we showed that nuclear CCN3 forms were able to modulate transcription in various eukaryotic cells.

Interestingly, a similar situation has just been reported for IGFBP proteins. Indeed, a strong transactivation domain (TAD) was identified in the N-terminal part of IGFBP2, IGFBP3 and IGFBP5 using a similar method [62], but this TAD is masked by inhibitory domains located in the central and carboxy-terminal parts of the protein [62]. IGFBP3 directly interacts with the Nuclear Retinoid X Receptor (RXR) and was shown to regulate gene transcription, using a RXR signaling reporter system [63,64]. IGFBP3 was indeed able to activate transcription via the RXR-binding element (RXRE) and down-regulate transcription via RAR-binding element (RARE), suggesting a co-activator/repressor role for IGFBP3 in transcription [63]. IGFBP3 interactions with RXR may lead to apoptosis in certain conditions (see below).

Regulation of transcription by nuclear RTKs was also initially investigated using such single hybrid systems [65]. To date, functional TADs were identified for various RTKs such as EGFR, ErbB2/HER2 and ErbB4/HER4 [39,46,47], and it appears that nuclear GFs and RTKs regulate the transcription of specific target genes. These observations open interesting fields of investigation for the CCN proteins. At the plasma membrane, CCN proteins are described as docking proteins [7,11,12]. Similarly, in the nucleus, CCN proteins may act as co-regulators, forming bridges between transcription factors and the basal transcription machinery. Along this line, the rpb7 subunit of RNA polymerase II has been identified as a CCN3 partner in a two-hybrid assay in yeast [34].

In summary, the above examples depict the variety of mechanisms of action of nuclear GFs and RTKs on the regulation of transcription. Some of them (such as the EGF/ErbB family, PRL, nucleolar FGF2) activate the transcription of specific target genes by interacting with specific transcription factors. Molecules like FGF1 and FGF2 can also interact with general co-regulators such as CBP and RSK proteins. Others (such as IGFBPs) seem to be able to either activate or inhibit gene transcription depending on their binding partners. They contain both TADs and TIDs (transinhibitory domains).

### Other actions in the nucleus

In addition to transcription, secreted proteins and RTKs that are transported to the nucleus seem also to directly regulate other important nuclear processes such as DNA repair and RNAs processing. Promoting DNA repair by ionization induces nuclear translocation of EGFR. This translocation results in activation of DNA-dependent Protein Kinase (DNA-PK), an important effector for the repair of DNA double-strand breaks [66]. These results suggest a role for nuclear EGFR in DNA repair and cell survival after irradiation.

Several studies suggest functions in the metabolism of the different RNAs. For example, PTHrP has been detected in the nucleus/nucleolus of a large variety of cells. PTHrP contains in its N-terminal part a consensus sequence found in numerous RNA binding proteins. PTHrP was found to directly bind several types of RNAs, suggesting a role in RNA metabolism [67].

Along the same line, nuclear FGF3 physically interacts with the Nucleolar FGF3-Binding Protein (NoBP; a.k.a. Ebp2p) and ribosomal protein S2 (rpS2) [68,69]. NoBP/Ebp2p is required for pre-rRNA processing and is essential for the synthesis of the 60S ribosomal subunit. RpS2 is a component of the small sub-unit of the ribosome. Nuclear FGF3 may therefore regulate ribosomal biogenesis.

HMW FGF2 (23 kDa) is associated with small nuclear Ribonucleoproteins particles (snRNPs) by physical interaction with the Survival of Motoneuron (SMN) protein, that functions as an assembly and recycling factor for the splicing machinery [70]. SMN can be located in the cytoplasm and in the nucleus. HMW FGF2–23 co-localises with SMN in the nucleus [70]. One of the subunits of the splicing factor 3a was identified as a binding partner for HMW FGF2–23 [71]. Electron microscopy studies revealed co-localization of FGFR1 with snRNPs in the nucleus of differentiating neurons but not proliferating neurons in cultures [51]. Therefore, nuclear FGF2/FGFR1 may be involved in mRNA processing during neuronal differentiation. Moreover, in as much as FGF2 is a neurotrophic factor for motoneurons (see below) and as SMN is deficient in patients with spinal muscular atrophy, a neurodegenerative disease, the FGF2-SMN complexes may be important for survival or degeneration of motoneurons.

The nuclear activities of secreted factors and RTKs cited above are summarized in Figure 2.

## Regulation of cell proliferation & differentiation

### Cell proliferation

Transcriptional targets and nuclear partners of nuclear GFs and RTKs are often involved in cell proliferation. For example, Cyclin D1, c-jun, c-Myc, c-fos, b-Myb, STAT3. It therefore seems that nuclear GFs and RTKs are linked to cell cycle progression and proliferation. In fact, correlation between nuclear localization of GFs/RTKs and proliferation has been established in various cell types a long time ago before the mechanisms of action were elucidated [72-75].

Nuclear EGFR has been detected for years in a variety of cell lines, normal and pathological tissues. Nuclear localization of EGFR was correlated with a high proliferative state in various tissues such as human placenta, human normal mouth mucosa, uterus of pregnant mice, or rat regenerative liver [46,76]. In this latter case, EGFR was indeed detected only in the nuclei of dividing hepatocytes, not in quiescent ones. Along the same line, in primary breast cancers, a positive correlation was established between high levels of nuclear EGFR and expression of cyclin D1 and Ki-67, two markers of active proliferation [46,77]. According with a role in cell proliferation, the translocation of ErbB1-EGF/Tumor Growth Factor-α (TGF-α) complexes to the nucleus seems to precede DNA replication [78,79]. Mutations introduced in the NLS of the EGFR-ligand SwGF result in the loss of its mitogenic activities.

Enhancement of *c-jun *expression by nuclear FGFR1, activation of rRNA transcription by nuclear FGFR1 and FGF2, and stimulation of DNA synthesis by nuclear FGF1 [25,80] are in favor of a role of nuclear FGFs/FGFRs in cell proliferation. It was indeed shown in various cell types that the accumulation in the nucleus of FGFs and FGFRs mostly takes place transiently during the G1 phase of the cell cycle and stimulates cell proliferation [17,28,54,55,81]. For instance, nuclear FGF2 maintains the RSK2 protein active during G1 phase, and FGF2 mutated in its sites of interaction with RSK2 loses 50% of its mitogenic activity [58]. Similarly, deletion of the NLS region in FGF1 considerably reduces FGF1 mitogenic activities [80]. Nuclear FGF2/FGFR1 may participate in the control of glial cell proliferation both in the embryo and also in the adult. FGF2 was indeed detected in the nucleus of rat brain astrocytes *in vivo *and *in vitro*, as well as in human astrocytes in culture [82,83]. FGF2 was observed in the nucleus of proliferating astrocytes, but was localized only in the cytoplasm in quiescent astrocytes [83]. FGFR1 was transiently detected in the nucleus of astrocytes during the G1 phase of the cell cycle. Glioma cells constitutively express high levels of nuclear FGF2 and FGFR1. Furthermore, increase in nuclear FGF2 and FGFR1 levels were observed after brain lesions and were associated with scar formation by proliferating astrocytes.

On the contrary to the above examples, nuclear FGF3 inhibits DNA synthesis and proliferation [84]. Interestingly, the nuclear functions of FGF3 are in opposition to that of secreted FGF3, which stimulates cell growth and transformation. Nuclear FGF3 acts through a physical interaction with NoBP and rpS2 proteins [68,69]. NoBP expression is associated with cell proliferation. It appears that nuclear FGF3 counteracts NoBP activities [68]. Similarly, rpS2 has been associated with up-regulation of proliferation and cancer cell growth. As for NoBP, FGF3 may also block rpS2 activities. FGF3 thus may inhibit cell proliferation by interfering with the ribosomal biogenesis.

Nuclear PTHrP is able to regulate both cell growth and cell death. Indeed, PTHrP entry into the nucleus induces proliferation of vascular smooth muscle cells [85], and nucleolar localization of PTHrP protects chondrocytes against apoptosis induced by serum starvation [86].

These few examples underline the variety of action of the nuclear GFs/RTKs on the cell cycle, by up-regulating or down-regulating cell proliferation and cell death.

### Cell differentiation during embryonic development and adult life

Nuclear FGF2 and FGFRs are involved in nervous system development. FGF2 is expressed in glial cells and certain neuronal populations. FGF2 acts as a neurotropic factor for a variety of central and peripheral neuronal types, acting either as a mitogen or a differentiating factor. It can promote neuronal survival, neuritogenesis and synaptic transmission. During rat nervous system development, both LMW and HMW FGF2s have been detected in the nuclear fraction of brain extracts [51]. More precisely, it was shown that FGF2 accumulated in the nucleus of cerebellar neurons as axonal growth took place. Once the synaptic connections were established, FGF2 nuclear labeling disappeared [87], though a recent publication reported that nuclear FGF2 was still detectable in certain neurons of adult mouse cerebellum [88]. FGFR1 has also been observed in the nucleus of developing rat brain cells and of a variety of neuronal cell lines. Treatments with stimuli such as Bone Morphogenic Protein 7 (BMP7) and cAMP, that induce neuronal-like differentiation, provoke nuclear accumulation of FGF2 and FGFR1 in various cell lines in culture. Moreover, nuclear FGFR1 is able to induce neuronal-like differentiation in a variety of neuronal cell lines. For example, FGFR1 expression mediates cAMP-induced cell cycle exit, neurite outgrowth and induction of neuron-specific genes in human neuronal progenitor cells (HNPCs) [51,81]. Interestingly, FGFR1 forms that remain at the plasma membrane fail to induce neuronal differentiation, and even block cAMP-induced neuronal differentiation [51,81]. Therefore, nuclear FGFR1 appears to be important for neuronal differentiation in the central nervous system. Increase in LMW and HMW FGF2s levels are detected in neurons and glial cells in peripheral nerves after injury. LMW, HMW FGF2s and FGFR1 accumulate in the nucleus of primary neonatal sympathetic neurons transiently transfected in culture [89]. In summary, it seems that nuclear FGF2s/FGFR1 are involved in neuronal differentiation in the central and peripheral nervous systems, and residual levels still detectable in adult, could be reactivated after lesion.

FGF2 and FGFRs also play critical roles during bone morphogenesis. Prostaglandins (PGs) are also involved in bone formation and resorption. It was very recently reported that PGF2α induced FGF2 nuclear translocation in rat osteoblasts and stimulated FGF2/FGFR2 internalization via clathrin-independent vesicles [90,91]. FGF2/FGFR2 complexes were subsequently found at the nuclear pore level, where they co-localized with IMPβ, suggesting a role for nuclear FGF2-FGFRs in bone formation.

The above examples reporting cellular functions for nuclear forms of secreted molecules often come from *in vitro *models. Like FGFR1 in NPCs, in Sertoli cells FGFR2 is detected in the nucleus of cells that withdraw from the cell cycle. Recently, the analysis of *fgf9*^-/- ^mice revealed that FGFR2 nuclear localization correlated with male sex determination in early gonads [92]. Nuclear FGFR2 coincided with the initiation of expression of the *Sry *gene, which determines the sex of the gonad, differentiating cells into Sertoli cells.

In adult, HMW FGF2s may be involved in wound healing. Pintucci and colleagues reported in a recent publication that, in an experimental injury model of vascular aortic smooth muscle cells, Platelet Derived Growth Factor (PDGF) rapidly induced expression of HMW FGF2, which accumulated in cell nuclei and nucleoli [93]. In contrast, PDGF did not induce LMW FGF2 expression, suggesting a specific role of nuclear HMG FGF2 in vascular remodeling after injury. Along the same line, in an *in vitro *model of wound healing using endothelial cells, VEGF was found to be rapidly internalized and specifically translocated to the nuclei of cells localized at the edges of the wound [94]. This phenomenon was accompanied in these cells by an increase in several wound healing related molecules such as integrin β3, factor VIII and tissue plasminogen activator, suggesting that VEGF nuclear accumulation may be involved in coagulation and fibrinolysis processes during repair after injury. VEGF was also endocytosed in cells away from the wound, but in this case, it was not imported to the nucleus, and no accumulation of repair related proteins was observed [94].

In summary, as for the classical extracellular pathways, nuclear GFs and RTKs can differentially regulate the cell proliferation and differentiation of target cell types, tissues and organs during embryogenesis. In adult, they may be involved in tissue repair after injury.

## Cancer

GFs and RTKs were detected in the nucleus of cancer cells a long time ago. In accord with their roles in cell cycle progression and proliferation, it is tempting to speculate that nuclear secreted factors and plasma membrane RTKs may sometimes be oncogenic, for example because of the deregulation of their shuttling between the cytoplasm and the nucleus in cancer cells. Some of their target genes or nuclear partners, such as Cyclin D1, c-jun, c-Myc, c-fos, b-Myb, COX2, STAT3 are known as key players in oncogenesis when they are deregulated. Overexpression of such genes can induce tumorigenesis. Recent studies showed that nuclear localization of secreted factors and RTKs in cancer cells is often associated with poor prognosis of survival. Furthermore a growing body of evidence suggests that nuclear secreted proteins and cell surface receptors are involved in tumor progression.

### Proliferation of cancer cells and tumor progression

PTHrP is expressed in breast epithelium and participates in normal mammary gland development and function. Most breast cancers secrete high level of PTHrP. Secreted PTHrP is in fact a prohormone that is cleaved into multiple peptides. The best characterized fragment (N-PTHrP) acts via cell surface PTHR1. The midregion of PTHrP (midPTHrP) binds another transmembrane receptor, is internalized and migrates to the nucleus via a bipartite NLS-dependent pathway [95]. High levels of MidPTHrP were detected in the nuclei of human non invasive MCF-7 and invasive MDA-MB231 breast cancer cells, but not in the nuclei of Hs578Bst non-tumor breast myoepithelial cells [95]. MidPTHrP increased proliferation of MCF-7 and MDA-MB231 cells, whereas it did not influence Hs578Bst growth [95]. A midPTHrP form that lacks the NLS and that remains in the cytoplasm did not increase proliferation of MCF-7 and MDA-MB231 cells [95], suggesting a role for nuclear PTHrP in breast cancer progression. Along the same line, ErbB4 overexpression in MCF-7 cells resulted in nuclear translocation of cleaved ErbB4, increased proliferation and anchorage-independent growth, and estrogen response element-mediated transcriptional activity [96].

Cancer cells frequently express HMW FGF2s. Increased levels of HMW FGF2s was observed during the transformation process *in vitro *[97,98]. Overexpression of HMW FGF2s leads to immortalization of primary cells in culture and allowed proliferation of immortalized cells in low-serum to achieve high cell density [99-101]. Cell-contact inhibition and mitotic activation were associated with nuclear accumulation of both LMW and HMW FGF2s in glioma cells [83]. Nuclear HMW (24 kDa) FGF2 facilitates cell survival *in vitro *and also during the establishment of metastases *in vivo *[102]. Taken together, these results suggest that HMW FGF2s might be involved in tumor progression. In some cases, the detection of high levels of HMW FGF2 in cell nuclei was even correlated with poor prognosis for patient survival (see below). FGFR1 accumulated in the nucleus in parallel with FGF2. Nuclear FGFR1 has been detected in both rapidly proliferating human medulloblastoma TE671 cells and slower proliferating glioma SF763 cells [103]. Furthermore, stable transfection of SF-763 glioma cells with either FGFR1 or HMW FGF2 resulted in nuclear localization of the receptor and the growth factor and in increased proliferation [51]. Along the same line, in breast tumors, FGF1 and FGFR3 are predominantly localized in the nuclei of epithelial cells, whereas they showed both nuclear and cytoplasmic localization in normal breast epithelium [104].

The CCN3 protein is detected in the nuclei of various cancer cell types, and this nuclear form of CCN3 is sometimes truncated, deprived of its amino-terminal part ([32,34] and unpublished data). The full-length secreted CCN3 exerts inhibitory effects on the proliferation of various human cancer cell lines, whereas an amino-truncated form deprived of the secretory peptide signal, was showing transforming activities on primary cells in culture (reviewed in [105-107]). We recently showed that such truncated forms of CCN3 are predominantly localized in the nucleus [5]. Therefore, the proliferation status of cells that express CCN3 may be the result of a subtle balance between the level of full-length secreted forms and nuclear amino-truncated forms of CCN3. A deregulation in this balance may result in tumorigenic events.

IL-1α is a pleiotropic cytokine involved in immune and inflammatory response, as well as in hematopoiesis. Furthermore, Il-1α stimulates growth of malignant cells such as gastric carcinoma cells, B-lymphoblasts, acute myelogenous leukemia cells, and Kaposi sarcoma cells. Secreted IL-1α is generated by proteolytic cleavage of an intracytoplasmic polypeptidic precursor by calpain protease. Whereas the carboxy-terminal part of the precursor is secreted and binds transmembrane IL-1 receptor, its N-terminal part (IL-1α propiece) is translocated to the nucleus via NLS-dependent mechanism [108]. Independently, secreted IL-1α bound to its transmembrane receptor can be internalized and also transported to the nucleus via a NLS located in the receptor. Interestingly, when stably expressed in glomerular mesangial cells, IL-1α propiece was able to induce anchorage-independent growth *in vitro *and very aggressive tumors *in vivo *when the IL-1α propiece-expressing cells were re-injected in Nude mice [108], suggesting that nuclear IL-1α propiece can act as an oncogenic protein.

### Association with bad prognosis, poor survival and resistance to anti-cancer therapies

Receptors of the EGFR/ErbB family have been detected in the nuclei of various human cancer types such as sarcoma, adrenocortical carcinoma, uterine cervix lesions, mouth cancer, and breast cancer. Several reports revealed positive correlations between nuclear localization of ErbB receptors and expression of target genes and nuclear partners in cancer cells: for example, ErbB2/HER2 and COX2 in choloangiocarcinoma, colon cancer and breast cancer [47]; also EGFR and cyclin D1/iNOS/STAT3 in breast cancers ([48], reviewed in [109]). Interestingly, the COX2 and iNOS enzymes produce inflammatory prostaglandins and nitric oxide respectively, molecules that emerge as targets for chemoprevention and chemotherapy. COX2 contributes to increased anti-apoptotic, pro-angiogenic, and metastatic potential in cancer cells, and deregulation of COX2 expression was associated with tumor progression, suggesting that COX2 overexpression induced by nuclear ErbB2/HER2 in cancer cells may be a marker of bad prognosis. Along the same line, the study of a large panel of breast carcinomas and oral squamous cell carcinomas established an inverse correlation between nuclear EGFR and patient survival, suggesting that nuclear EGFR accumulation may be indicative of poor clinical outcome [77,110]. This observation may, in part, be due to increased proliferative capacity in cancer cells that exhibit nuclear EGFR. Nuclear ErbB4/HER4 was also associated with poor prognosis in breast cancer, in contrast to membrane bound ErbB4/HER4 [96].

Interestingly, in human bronchial and breast tumor cells, blocking the ionization-induced nuclear import of EGFR by the use of anti-EGFR antibodies increased radio-sensitivity of treated cells, by sequestering DNA-PK in the cytoplasm, complexed to EGFR [111]. Preclinical studies showed that anti-EGFR antibodies enhanced anti-tumor effects of irradiation, suggesting that combining immunotherapy to radiotherapy and chemotherapy may be successful in anti-tumor therapies for cancers exhibiting EGFR nuclear localization [111].

EGFR does not only bind extracellular EGF, but also Heparin Binding Epidermal Growth Factor-like (HB-EGF). HB-EGF can be internalized and transiently translocated to the nucleus during late G1 and S phases of the cell cycle. Binding and triggering nuclear export of Promyelocytic Leukemia Zinc Finger protein (PLZF), a transcriptional repressor of the *cyclin A *gene, HB-EGF promotes cell cycle progression into the S phase [112]. HB-EGF is generated by proteolytic cleavage of the transmembrane precursor proHB-EGF. The intracytoplasmic part of proHB-EGF can also migrate to the nucleus, and thereby alters cell cycle in EGFR-independent manner [113]. Full-length proHB-EGF was also detected in the nuclei of human TCCSUP bladder cancer cells, and correlated with poor prognosis in bladder cancers [114,115]. Furthermore, Reactive Oxygene Species (ROS), metabolites that are overproduced in cancer cell as a result of transformation process, induced nuclear export of proHB-EGF in TCCSUP cells. Exported proHB-EGF was then cleaved by a metalloproteinase, HB-EGF was secreted in the extracellular compartment, where it activated transmembrane EGFR, resulting in stimulation of cell proliferation and partial protection from cisplatin and oxidative stress-induced apoptosis [115]. These results support the idea of an autocrine loop leading to cell proliferation and protection from apoptotic stimuli in bladder cancer cells [115]. Taken together, these results show that various nuclear forms of HB-EGF can promote cancer progression by distinct mechanisms.

In human astrocytic tumors cells, high levels of nuclear HMW FGF2s have been correlated with poor prognosis [116]. Furthermore, overexpression of HMW FGF2s has been observed in various human cancer cells of patients resistant to radiotherapy. It has even been shown that overexpression of HMW FGF2s in HeLa cells protected them from irradiation by directly up-regulating the expression of the DNA-PKcs, which encodes one of the sub-unit of the DNA-repair DNA-PK enzyme [117]. As for EGF and HB-EGF, targeting nuclear HMW FGF2s may generate new anti-cancer therapies for patients resistant to classical protocols.

### Promotion of apoptosis of cancer cells

IGFBPs bind Insulin-like Growth Factors (IGFs) in the extracellular compartment and modulate their mitogenic effects, but IGFBPs can also act on cell proliferation and apoptosis via IGF-independent mechanisms. IGFBPs modulate proliferation and survival of various tumor cell types including osteosarcoma cells. The IGFBP3 protein, which is encoded by a tumor suppressor gene, exhibits anti-proliferative and pro-apoptotic functions, both in IGF-dependent and IGF-independent pathways. IGFBP3 has been detected in the nuclei of human lung, breast and prostate cancer cells. IGFBP3 induces apoptotic cell death in human colon carcinoma cells and breast cancer cells. Furthermore, nuclear IGFBP3 is able to enhance apoptosis in U-2 OS osteosarcoma cells, in a caspase-dependent mechanism [43]. In agreement with a proapototic role of nuclear IGFBP3, endogenous IGFBP3 is detected only at very low levels in the nuclei of U-2 OS cells [43]. Since a proteasome-dependent degradation of nuclear IGFBP3 has been described in U-2 OS cells, IGFBP3 may therefore be subject to a rapid turn over by proteolysis in the nucleus of cancer cells [43]. IGFBP3 and RXR ligands have additive effects on apoptosis of human prostate cancer cells both *in vitro *and *in vivo *[118]. RXR α is even essential for apoptotic functions of IGFBP3. In as much as nuclear IGFBP3 is able to modulate transcription via RXR binding sites (see above), nuclear IGFBP3 may cooperate with retinoic receptors to induce gene transcription leading to apoptosis in cancer cells [63].

Though Il-1α propiece has been described as an oncogenic protein (see above), more recently, nuclear IL-1α propiece was shown to induce apoptosis in a large panel of cancer cell lines of several origins such as lung, colon, central nervous system, blood, skin, kidney, breast, ovary, whereas it did not in non malignant primary cells [119]. IL-1α propiece co-localized in the nucleus with several components of the spliceosome machinery and provoked a decrease in anti-apoptotic alternate form of Bcl-X. As a consequence a change in the balance between anti- and pro-apoptotic forms of Bcl-X occurs in favor of the pro-apoptotic form [119]. These data suggest that nuclear IL-1α propiece may induce apoptosis in cancer cells by modifications in RNA processing. Taken together, these results underline a dual role for nuclear IL-1α depending on cellular context.

In summary, nuclear localization of GFs and RTKs of the FGF and EGF/Erb families in cancer cells seems to be associated with a bad prognosis for patient survival. At least two reasons can explain this correlation. The first one is their role in the promotion of cancer cell proliferation and transformation, which favors tumor progression. The second one is in the resistance to radiotherapy that they appear to induce because of their potential role in DNA repair. It is worth noting that, in agreement with its role in cell proliferation, FGF1 has been detected in the nucleus of certain cells within inflammatory arthritic joints in the synovium from patients with rheumatoid arthritis [120]. This disease is characterized by massive proliferation of synovial connective tissues and invasive destruction of periarticular bone and cartilage. These data suggest a role for nuclear GFs in pathologies other than cancers. By contrast, inhibiting cell proliferation and promoting apoptosis of cancer cells, other secreted factors such as IGFBPs may be markers of good prognosis.

## Conclusions and future considerations

The nuclear localization of secreted proteins and plasma membrane receptors has been known for more than 15 years, but their nuclear translocation and functions has remained largely unknown until recently. The number of studies describing diverse physiological functions for nuclear ligands and their receptors has grown rapidly in the literature since 2004/2005. Though the discovery of transcriptional activity of nuclear EGFR on cyclin D1 promoter in 2001 by Lin and colleagues in Nature Cell Biology [46] lead to a comment entitled "EGF receptors as transcription factors: ridiculous or sublime?" in the same volume [121], the physiological functions of nuclear secreted ligands and their receptors could no longer be subject to controversy.

**Figure 4:**
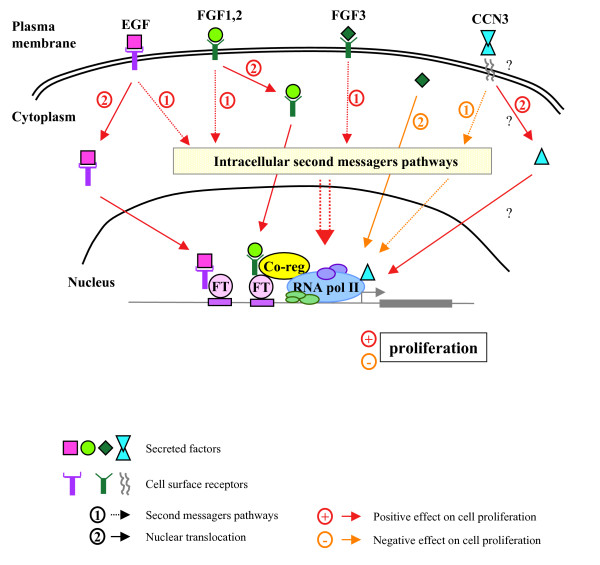
**Possible mechanisms of action for secreted protein function in cell proliferation**, either by intracellular second messengers pathways or by nuclear import. FT: transcription factor; Co-reg: Co-regulator.

The classical view on the modes of action of the secreted molecules has evolved during the last decade. It appears today that GFs act on cells by several distinct mechanisms. Activated RTKs by ligand binding can induce signals inside the cell through several second messenger pathways depending on the cellular context. In addition, receptor-bound GFs can be internalized by endocytosis and translocated to the nucleus (Figure 4). FGF1, FGF2 and FGF3 are known to act both extracellularly and intracellularly. Although FGF2 was shown to activate rRNA transcription in both ways, secreted FGF3 and nuclear FGF3 exhibit opposite functions on cell proliferation. The case of CCN3 resembles the one described for FGF3. Therefore, it appears that secreted forms and nuclear forms of GFs can act either in concert or in opposition (Figure 4).

In the nucleus, secreted factors and receptors regulate the transcription of specific target genes by direct binding to transcription factors and general coregulators. They could also be involved in other processes such as DNA replication, DNA repair and RNA metabolism. Their target genes and protein partners often become oncogenic when they are deregulated. Therefore alteration of nuclear shuttling of secreted molecules and cell surface receptors may result in the development of pathologies of cell proliferation. A growing body of evidence correlates nuclear localization of GFs and RTKs with cancer progression in various organs in human. The presence of some of them in the nucleus even seems to provoke resistance to radiotherapy. Targeting nuclear import of the secreted molecules and their receptors opens new areas for investigation for future anti-cancer therapies.

## Abbreviations

GF: Growth Factor; RTK: Receptor-Tyrosine Kinase; NLS: Nuclear Localization Signal; NES: Nuclear Export Signal; IMP: Importin; EXP: Exportin; LMW: Low Molecular Weight; HMW: High Molecular Weight; TAD: Transactivation Domain; DBD: DNA binding domain; FGF: Fibroblast Growth Factor, EGF: Epidermal Growth Factor; VEGF: Vascular Endothelial Growth Factor; IL-1: Interleukin-1; IFNγ: Interferon-γ; GH: Growth Hormone; PRL: Prolactin; IGFBP: Insulin Like Growth Factor Binding Protein; CCN: Cyr61-Ctgf-Nov; SwGF: Schwannoma-derived growth factor; PTHrP: Parathyroid Hormone-related Protein; GFP: Green Fluorescent Protein; STAT: Signal Transducer and Activator of Transcription; CypB; Cyclophilin B protein.

## Competing interests

The author(s) declare that they have no competing interests.
